# Pre-activated macrophage membrane-encased aggregation-induced emission featuring nanoparticles: a novel possibility for tuberculosis treatment

**DOI:** 10.1038/s41392-024-01855-8

**Published:** 2024-06-26

**Authors:** Qiang Cai, Yihao Tian, Quazi T. H. Shubhra

**Affiliations:** 1 Department of Neurosurgery, Renmin Hospital of Wuhan University, Wuhan, China; 2 Department of Human Anatomy and Histology and Embryology, School of Basic Medical Sciences, Wuhan University, Wuhan, China; 3 Institute of Chemistry, University of Silesia in Katowice, Szkolna 9, Katowice, Poland

**Keywords:** Biotechnology, Drug discovery, Diseases

In a recent study published in *Nature Nanotechnology*, Li et al. introduce the synthesis of a novel photosensitizer with unique aggregation-induced emission (AIE) characteristics.^1^ They microencapsulated this photosensitizer inside engineered nanoparticles (NPs) coated with pre-activated macrophage cell membranes (CMs), an innovative approach marking a significant leap in nanomedicine by offering a pathway to potent tuberculosis (TB) treatments that bypass antibiotic resistance with unparalleled precision.

TB, a life-threatening chronic infectious disease caused by *M. tuberculosis*, remains one of the most formidable challenges in global health, being the 13th leading cause of worldwide mortality and the 2nd deadliest infectious disease after COVID-19. The effectiveness of traditional TB treatments has been limited by their decreased efficacy, high toxicity, and the growing problem of antibiotic resistance. This underscores the urgent need for new therapeutic approaches. Recent advancements in nanotechnology have unlocked the potential for anti-TB nanotherapeutics. However, the specific targeting of the pathogen and demonstration of efficacy in mammalian models remained unexplored areas until the exciting work by Li et al.

**Fig. 1:**
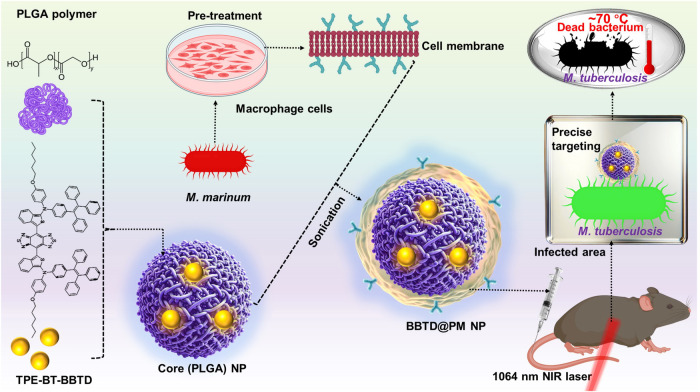
Pre-activated macrophage membrane-coated PLGA nanoparticles for targeted tuberculosis therapy. Pre-treatment amplifies macrophage receptor expression for specificity towards *M. tuberculosis*. Encapsulating the novel photosensitizer TPE-BT-BBTD within PLGA cores and enveloping with pre-activated MCMs creates BBTD@PM NPs, aimed at precise imaging and phototherapy under NIR-II laser irradiation. This strategic design focuses on localizing and eradicating tuberculosis with minimal impact on adjacent healthy tissues. Some components of this figure were created with BioRender.com

AIE luminogens, discovered by Prof. Ben’s team in 2001, exhibit a unique property: they are non-emissive in a dispersed state but emit light upon aggregation, making them highly suitable for disease bioimaging.^2^ While the combination of AIE compounds with antibiotics in engineered NPs could offer a synergistic theranostic strategy for TB, Li et al. advanced this concept further. They synthesized a novel photosensitizer (TPE-BT-BBTD) endowed with AIE properties, enabling the dual functionality of photothermal therapy (PTT) and bioimaging within a single agent. This compound, synthesized through a palladium-catalyzed two-step process, is excited at near-infrared II (NIR-II) wavelengths (980 and 1064 nm), enabling deep tissue penetration. Addressing the hydrophobic nature of TPE-BT-BBTD, a challenge for effective delivery to target sites, they microencapsulated the compound using poly(lactic-co-glycolic acid) (PLGA) polymer via nanoprecipitation (Fig. 1).

Since the early last decade, CM coating has emerged as a transformative biomimetic technology in nanomedicine.^3,4^ This technique involves enveloping NPs with CM, allowing their surface to obtain the original cell’s proteins, lipids, and carbohydrates. Consequently, coated NPs can seamlessly integrate into biological systems to show augmented accumulation in disease sites. Notably, macrophage CM (MCM) coatings have shown exceptional ability in recognizing pathogens such as viruses, bacteria, and fungi, thereby enhancing the effectiveness of nanotherapeutics against a range of pathogens.^5^ In their study, the authors innovatively employed pre-activated membranes (PMs) from *M. marinum*-treated RAW 264.7 macrophage cells, exhibiting high levels of TB-specific receptors. By wrapping TPE-BT-BBTD-containing PLGA NPs with these PMs, they fabricated BBTD@PM NPs. The brilliance of this methodology lies in its dual-targeting mechanism, which takes advantage of both the enhanced permeability and retention (EPR) effect and specific receptor-ligand interactions for direct targeting of granulomas and internal *M. tuberculosis*. Such targeted delivery is instrumental in achieving higher concentrations of therapeutic agents at the site of infection, thereby enhancing treatment efficacy while minimizing systemic toxicity.

It is very much appreciated that the authors presented the transcriptomic analysis of *M. marinum*-stimulated macrophages, providing valuable insights. This analysis revealed significant morphology and gene expression changes, indicating a strong immune response. Extended exposure led to cell death, with pathway analysis highlighting the upregulation of TB-specific and Toll-like receptor (TLR) pathways, signifying intensified inflammatory response. Notably, the expression of TLR2, TLR4, and TLR6 was significantly increased, underscoring their critical roles in combating *M. marinum* and their potential as targets in TB therapy. Importantly, these TLRs (TLR2, TLR4, TLR6) were present on BBTD@PM NPs, confirming the successful CM coating for precise TB targeting. These NPs also demonstrated key characteristics for PTT: a small size (less than 100 nm size) and a high negative zeta potential (−21.3 mV), ensuring long circulation and stability (over 15 days). Moreover, their photothermal conversion efficiency of 33.8% is well-suited for effective PTT. Under NIR laser irradiation, they reached a therapeutic temperature of ~70 °C within 10 min, indicating their potential for pathogen destruction. Regarding biotoxicity, the BBTD@PM NPs exhibited minimal toxicity in vitro, showing no adverse effects on human erythrocytes or normal cell proliferation. Moreover, in vivo studies revealed no significant weight loss in mice and no acute or chronic damage to the hematological system, liver, or kidneys after treatment with these NPs.

The BBTD@PM NPs exhibited superior targeting and bactericidal effects against *M. tuberculosis* compared to RBC-coated NPs due to TLRs on PMs. With a high specific affinity for the H37Ra strain, these NPs showed intense photothermal action when exposed to 1064 nm laser irradiation, leading to a significant decrease in bacterial colonies. Notably, the NPs were nontoxic to *M. tuberculosis* without irradiation but effectively killed most bacteria upon irradiation. Their NIR-IIb imaging capabilities allowed for deep tissue penetration (up to 9 mm) and precise targeting of tuberculous granulomas in lungs, surpassing imaging using traditional dyes like indocyanine green. In vivo studies on H37Ra-induced pulmonary TB in mice demonstrated that BBTD@PM NPs significantly lowered bacterial counts after irradiation. Histopathological analysis revealed reduced inflammation and enhanced lung tissue repair, outperforming the effectiveness of standard anti-TB antibiotic treatments.

The work undoubtedly sets the stage for future investigations into infectious diseases using cutting-edge nanotechnology. While their innovation offers a fresh perspective on TB management, the journey ahead calls for a deeper inquiry into several key areas. Due to biosafety considerations, the pragmatic choice of *M. marinum* underscores the need for subsequent studies directly involving *M. tuberculosis* to improve clinical applicability. Both active and passive targeting strategies present challenges for TB treatment. The EPR effect, a cornerstone of passive targeting, may exhibit variability between TB patients, potentially compromising consistent drug delivery. Active targeting strategies may face hurdles due to the heterogeneity of TB lesions and the variable expression of receptors among different TB strains. The failed phase 2 trial (NCT02479178) of the actively targeted nanoparticle BIND-014 exemplifies these limitations. This underscores the imperative for efficient TB targeting strategies that effectively navigate the complexities of both targeting mechanisms to optimize therapeutic outcomes. Additionally, evaluating the practicality of the 9-mm penetration depth in a clinical context, especially considering the human chest wall’s greater thickness, is crucial to validate the efficacy of NIR-IIb for TB diagnosis and treatment. The potential to apply this photosensitizer in future cancer treatment by dual-targeting strategy, specifically through cancer cell membrane coatings, is highly anticipated.^3^ Investigating the approach’s applicability to extrapulmonary TB is eagerly awaited. Future endeavors should focus on evaluating these NPs in diverse animal models that accurately reflect human disease pathophysiology, extending their application beyond tuberculosis to fully harness the PTT and/or imaging capabilities of these AIE-based nanoparticles. Additionally, co-delivery of this photosensitizer with traditional TB medications using the same NPs could lead to lower dosages and reduced resistance, warranting thorough examination. Overcoming challenges in scalability and manufacturability and ensuring long-term safety will be key milestones in the journey towards clinical implementation.

In conclusion, this study marks a pivotal step forward in TB therapy, establishing a solid foundation for the development of innovative treatments. The insights derived from this work promise to guide future research endeavors toward developing more efficacious therapeutic strategies for TB.
